# The impact of the COVID-19 pandemic on the provision of endovascular thrombectomy for stroke: an Irish perspective

**DOI:** 10.1007/s11845-023-03314-9

**Published:** 2023-02-16

**Authors:** Conor Brosnan, David Brennan, Conor Reid, Sarah Power, Alan O’Hare, Paul Brennan, John Thornton, Matthew Crockett

**Affiliations:** 1 Department of Neuroradiology, Beaumont Hospital, Beaumont, Dublin 9, D09 V2N0 Ireland; 2 Faculty of Radiologists, RCSI, Dublin, Ireland; 3 National Thrombectomy Service, Beaumont Hospital, Dublin, Ireland

**Keywords:** COVID-19, Large vessel occlusion, Stroke, Thrombectomy

## Abstract

**Background:**

The COVID-19 pandemic produced unprecedented challenges to healthcare systems. These challenges were amplified in the setting of endovascular thrombectomy (EVT) for large vessel occlusion strokes given the time-sensitive nature of the procedure.

**Aims:**

To assess the impact of the COVID-19 pandemic on service provision at the primary endovascular stroke centre in Ireland.

**Methods:**

A retrospective review of the National Thrombectomy Service database was performed. All patients undergoing EVT from 1 January to 31 December inclusive of 2019 to 2021 were included. Patient demographics, functional outcomes and endovascular treatment time metrics were recorded.

**Results:**

Data from 2019, 2020 and 2021 were extracted. Three hundred seven thrombectomies were performed in 2019 and 2020; this number increased to 327 in 2021. Median time from arrival to groin puncture for thrombectomy was 64 min in 2019, increasing to 65 min in 2020. In 2021, this decreased to 52 min. Median time taken from groin puncture to first perfusion remained stable from 2019 to 2021 years at 20 min. Total duration of emergency thrombectomies reduced from 32 min in 2019 to 27 min in 2020. This increased to 29 min in 2021.

**Conclusions:**

Despite the myriad of challenges presented by the pandemic, service provision at the primary Irish ESC, and the referring hospitals, has proven to be robust. Procedural time metrics were maintained whilst the expected reduction in number of EVTs performed did not materialise, there actually being a significant increase in number of EVTs performed in the pandemic’s second year.

## Background

Novel coronavirus severe acute respiratory syndrome coronavirus 2 (SARS-CoV-2) and the coronavirus disease (COVID-19) have had a huge impact on non-COVID-19-related care in hospitals. To date, there have been over 650 million documented cases and over 6.5 million reported mortalities [1]. Literature has demonstrated an association between COVID-19 and acute stroke, with an incidence of 1.4% of those affected with the disease and almost 80% of COVID-19 patients with acute ischaemic stroke (AIS) have been shown to have a large vessel occlusion (LVO) pattern [2]. Endovascular therapy (EVT) has been proven to be the gold standard management of LVO strokes [3]. Prompt management with EVT is imperative, with small reductions in treatment delays providing marked improvements in long-term functional outcomes [4, 5]. The COVID-19 pandemic presented unprecedented challenges to global healthcare. Testing, contact precautions, use of personal protective equipment and infection control procedures may all impede expedient management of patients with AIS secondary to LVO.

The first case of COVID-19 in Ireland was confirmed on 29 February 2020, and over 1.6 million cases and 8000 deaths have since been recorded [1, 6]. When the pandemic initially took hold, an unprecedented impact on service provision swept across the nation, with significant delays in almost all non-COVID-19-related care as a nationwide ‘lockdown’ was imposed [7]. Challenges included staff redeployment, increased staff sick leave, delays in patient presentation and infection control measures. Spikes in case numbers also threatened to overwhelm the healthcare system and deplete resources. Despite numerous guidelines and recommendations published to advise management considerations with COVID-19, significant concerns were expressed in relation to potential hold up of treatment in AIS with LVO [8–11].

This study aims to assess the impact of COVID-19 on service provision at the primary endovascular stroke centre (ESC) in Ireland. EVT time metrics constitute the primary outcome measure in this study. Numerous secondary outcomes were also investigated, including the total number of referrals and patient outcomes.

We hypothesised that the total number of AIS patients referred to the thrombectomy centre with LVO, as well as the number of EVTs performed, would reduce following the onset of the pandemic. This would be due to a combination of delayed patient presentation to the hospital, local hospital workflow issues and the logistical challenges of interhospital transfers. We also hypothesised that EVT procedure time metrics would disimprove because of delays associated with COVID infection control measures amongst other factors.

## Methods

Beaumont Hospital is Ireland’s national neurosciences centre and primary endovascular stroke centre (ESC), providing emergency EVT services for patients with AIS resulting from LVO to the majority of the country’s population. Until early 2021, a national 24-h thrombectomy service was provided solely by Beaumont Hospital with Cork University Hospital providing daytime cover for the majority of the South of the Republic of Ireland. In April 2021, Cork University Hospital commenced 24-h emergency EVT provision [12].

In Ireland, patients presenting with suspected stroke outside of the geographical catchment area of an ESC are assessed, imaged and treated with intravenous thrombolysis at the nearest PSC. Candidates for EVT identified on imaging are discussed directly with an ESC regarding suitability for consideration of EVT. Patients were transferred for EVT from 21 sites in 2020 and 20 sites in 2021, in addition to direct presentations to our institution [12–14].

Suspected stroke cases presenting to PSCs undergo a clinical examination and subsequent radiological investigation including initial non-contrast CT followed by single or multiphase CT angiography (CTA). In select cases, CT perfusion may be also performed, although this is only available in a few centres in the country. EVT eligibility is based on National Thrombectomy Service guidelines derived from the best evidence [15, 16]. Patients are assessed on a case-by-case basis, with general inclusion criteria including all patients with an LVO presenting within 24 h of symptom onset or last known well time, with an ASPECTS score of > 5. After the commencement of intravenous thrombolysis, if appropriate, all cases eligible for EVT are discussed immediately with the interventional neuroradiology service of the ESC. Decisions for transfer are made on a case-by-case basis. Patients suitable for further management are transferred immediately by paramedics, accompanied by medical and nursing staff. Repeat assessment is performed on arrival by the stroke team at the ESC. Repeat imaging may be performed in some instances prior to transfer to the neurointerventional suite for EVT.

Numerous measures were taken to mitigate the spread of COVID-19 to healthcare workers providing EVT to patients with AIS and LVO. Necessary efforts were also made to reduce possible delays in providing treatment. Implemented measures followed the most up-to-date international guidelines available at the time [8, 17].

All patients presenting directly to our centre were tested immediately on arrival for COVID-19. Testing was performed on all patients presenting to external PSCs prior to transfer. Initial reverse transcriptase polymerase chain reaction (RT PCR) testing was replaced by rapid COVID-19 GeneXpert testing. For cases returning a negative result, standard infection precautions were followed. Patients with a positive test and those without a negative result were treated as positive. Our centre houses two neuroangiographic suites, with a dedicated COVID-19 suite in the setting of two available rooms. To expediate management, all procedural equipment for standard EVT was pre-prepared outside both thrombectomy suites in an expectant fashion.

For COVID-19-positive cases, all unnecessary objects were removed from the angiographic suite prior to the procedure, and all surfaces were covered with aseptic drapes. Staff for each procedure were kept to a minimum with a single nurse, radiographer and consultant interventional neuroradiologist (and a single interventional neuroradiology fellow during normal working hours) present. Doors of the suite were taped closed during the procedure. Additional personal protective equipment was used with a double-glove technique, N-95-approved face masks, shoe covers and face shields worn to reduce spread.

### Study periods, patient population and outcomes

A retrospective review of the prospectively maintained National Thrombectomy Service database in Beaumont Hospital was performed. All patients with AIS and LVO presenting to our centre or transferred from external sites for thrombectomy between 1 January 2019 and 31 December 2021 were included. Data obtained was broken down into three separate study periods, 1 January 2019 to 31 December 2019, representing the year prior to COVID-19, 1 January 2020 to 31 December 2020, representing the initial impact of COVID-19, and 1 January 2021 to 31 December 2021 to represent continued care and the response to COVID-19.

Baseline demographics were acquired for 2019, 2020 and 2021, including patient age and sex (detailed patient demographics are displayed in Table 1). The number of patients with AIS secondary to LVO and presentation to ESC or external PSC were documented. Degree of neurological deficit (objectively measured by the National Institutes of Health Stroke Scale (NIHSS)) and basic image findings such as Alberta Stroke Programme Early CT score (ASPECTS) and occlusion site were also recorded.

The primary outcome of the study was time of arrival at ESC to time of groin puncture (door-to-puncture time), which was assessed as a continuous variable. Several other time metrics were also analysed over the study period including median procedure duration and median time from groin puncture to recanalisation, median door-to-imaging time, median door-to-intravenous thrombolysis time and median time of symptom onset/last seen well to presentation, groin puncture and recanalisation. Measures of response to thrombolytic therapy (using the ‘thrombolysis in cerebral infarction (TICI)’ scoring system) and patient functional outcomes (using the ‘modified Rankin scale (mRS)’ scoring system) were also assessed.

TICI scores were recorded by the interventional neuroradiology consultant performing the procedure for all anterior circulation strokes. A second, independent interventional neuroradiologist reviewed imaging and ascribed a TICI score to each case prior to entry into the retrospective database. Any discrepancies were resolved by a third, senior interventional neuroradiologist. Rates of 2b, 2c and 3 are considered good outcomes [18–20]. Expected rates internationally are 80% for TICI 2b-TICI3 inclusive [12].

mRS scores were recorded routinely 90 days after presentation. A good functional outcome was taken to be an mRS score of ≤ 2 or the ability to perform basic activities of daily living without a problem [21, 22].

## Results

Over the course of the study period, a total of 940 patients underwent EVT at Beaumont Hospital. Eight hundred six patients were transferred from PSCs and 158 patients presented directly to Beaumont Hospital. Three hundred seven thrombectomies were performed in 2019 and 2020 and 327 were performed in 2021. The total number of patients transferred for thrombectomy was 357 in 2019 and 2020, with an increase to 393 in 2021. A total of 167 patients were transferred to the ESC but deemed unsuitable for thrombectomy upon arrival over the course of the study period. This included 51 patients in 2019, 50 in 2020 and 66 in 2021.

The median age of presentation was 73. An equal number of males and females presented in 2019. More females presented than males in 2020 (155 and 152 respectively). In 2021, more males presented than females (177 and 150 respectively). The median NIHSS on arrival to ESC was 15 in 2019, 16 in 2020 and 14 in 2021. The median pre-intervention ASPECTS score was 9 in all 3 years examined. Patient demographics are displayed in Table 1.

**Table 1:** Patient demographics

**Demographics**	Total (*n* = 940)	2019 (*n* = 306)	2020 (*n* = 307)	2021 (*n* = 327)
**Median age**	73	73	72	73
**Male:female**	482:458	153:153	152:155	177:150
**Median arrival NIHSS**	15	15	16	14
**Median pre-intervention ASPECTS**	9	9	9	9
**% thrombolysed**	39% (364/938)	42% (128/304)	43% (132/307)	32% (105/327)
**Occlusion site**				
**Multiple occlusions**	136 (14%) of patients had > 1 occlusion	57 (18%) of patients had > 1 occlusion	21 (7%) of patients had > 1 occlusion	58 (14%) of patients had > 1 occlusion
**Total occlusions**	1127	373	330	424
ACA	17	3	5	9
Basilar/PCA/vert	79	27	17	35
ICA cervical/carotid T/L	246	100	65	81
MCA (M1 + M2)	785	243	243	299
Total considered for thrombectomy	1107	357	357	393
Thrombectomy candidates transferred from PSC	806	266	257	283
Thrombectomy candidates presenting to ESC	134	40	50	44
Already in ESC*	24	10	7	7
Total number of thrombectomies	941	307	307	327
Unsuitable for thrombectomy	167	51	50	66

Most patients managed with EVT had a middle cerebral artery (MCA) occlusion, which accounted for 69% (785/1127) of total occlusions in the study period. Sixty-five percent (243/373) of occlusions were in an MCA distribution in 2019 with 74% (243/330) and 71% (299/424) in the MCA 2020 and 2021. The next most common site of occlusion was the internal carotid artery (ICA), which made up 22% (246/1127) of total occlusions overall. ICA occlusions accounted for 27% of all occlusions in 2019 (100/373) and 19% in 2020 (65/330) and 2021 (81/424). Seven percent (79/1127) of occlusions were in the posterior circulation, which made up 7% (27/373) in 2019, 5% (17/330) in 2020 and 8% (35/424) in 2021. Anterior cerebral artery (ACA) occlusions comprised 2% (17/1127) of total occlusions (1% (3/373) in 2019, 2% (5/330) in 2020 and 2% (9/424) in 2021. In total, 14% (136/1127) of patients had more than one occlusion. In 2019, 18% (57/373) of patients had over one occlusion; this figure dropped to 7% (21/330) in 2020 and increased again in 2021 to 14% (58/424).

Patient demographics and occlusion site figures were comparable across all three study periods. There was no statistically significant difference in the degree of the neurological deficit between 2020 and 2021 (mean presenting NIHSS 16 and 14 respectively) and the median pre-intervention ASPECTS score did not vary between either year (9).

### Primary outcome

The median door-to-groin puncture time increased from 15 min in 2019 to 18 min in 2020 and 2021. For patients transferred to ESC from PSCs, door-to-puncture time was 14 min in 2019 and 15 min in 2020 and 2021. The door-to-puncture time for those presenting directly to ESC was 64 min in 2019 and 65 min in 2020. This figure dropped to 51 min in 2021.

### Secondary outcomes

Numerous other time metrics were examined in this study. The median time from symptom onset/time last seen well to presentation was seen to increase from 115 min overall in 2019 to 157 min in 2020 and 156 min in 2021. For patients presenting directly to ESC, this figure was 100 min in 2019, increasing to 165 min in 2020 and 157 min in 2021. The median time was higher in those presenting to PSCs at 115 min in 2019 and was recorded to be 150 min in 2020 and 154 min in 2021.

The median time from presentation to commencement of radiological investigation (door-to-imaging time) was 22 min overall in 2019 and 22 min in 2020. This increased to 24 min in 2021. At ESC, the door-to-imaging time reduced from 21 min in 2019 to 20 min and 18 min in 2020 and 2021 respectively. Conversely, the door-to-imaging time increased slightly in patients presenting to other PSCs between the three study periods observed (22 min in 2019, 23 min in 2020 and 25 min in 2021).

Overall, 39% (364/938) of patients received intravenous thrombolysis. Forty-two percent (128/304) received IV thrombolysis in 2019 and 43% (132/307) in 2020. This figure dropped to 32% (105/327) in 2021. The time taken to institute thrombolytic therapy (door-to-needle time) was observed to reduce across the length of the study (50 min overall in 2019, 48 min in 2020 and 42 min in 2021), with shorter door-to-needle times seen in both ESC and other PSCs from year to year (44 min, 43 min and 28 min and 54 min at ESC and 50 min and 49 min at PSCs in 2019, 2020 and 2021 respectively).

The median time from symptom onset/time last seen to groin puncture was observed to increase from 2019 to 2020 and subsequently decrease in 2021. At ESC, the median time rose from 192 min in 2019 to 242 min in 2020 and back to 160 min in 2021. There was less variability in this metric at other PSCs with median time rising from 335 min in 2019 to 376 min in 2020 and 355 min in 2021. Overall timing mirrored trends of individual PSCs at 322 min, 349 min and 347 min in 2019, 2020 and 2021 respectively.

Regarding time metrics directly pertaining to EVT, there was little variability seen for overall procedure time or time from groin puncture to recanalisation. Procedure duration was observed to be 32 min in 2019, 27 min in 2020 and 28 min in 2021 (37 min, 31 min and 37 min in 2019, 2020 and 2021 respectively for those directly presenting to ESC and 31 min, 26 min and 27 min in 2019, 2020 and 2021 for those transferred from other sites). Time from puncture to recanalisation reduced from 20 min in 2019 to 19 min in 2020 and 20 min in 2021 overall (26 min, 22 min and 22 min in those directly presenting to ESC and 19 min, 19 min and 20 min in those transferred from other sites.

Regarding the technical success of thrombectomy procedures performed, TICI 2b-3 recanalisation rates were reported in 91% (257/281) of cases in 2019. This figure increased to 94% (272/289) in 2020 and 96% (289/302) in 2021. mRS scores at 90 days post-thrombectomy were used to assess the level of function, with 45% (136/302, 105/234) of patients reported to have a good outcome (mRS ≤ 2) in 2019 and 2020 and 48% (151/315) in 2021 (Table 2).

**Table 2:** Time metrics and functional outcomes for those undergoing thrombectomy

**Outcomes**	**2019**	**2020**	**2021**
**Symptom onset/last seen well to presentation, median minutes (IQR)**			
ESC	100 (65–362) (*n* = 30)	165 (68*–*319) (*n* = 43)	157 (64*–*507) (*n* = 36)
Transfers	115 (65*–*369) (*n* = 234)	150 (78*–*380) (*n* = 215)	154 (***) (*n* = 240)
Overall	115 (65*–*371) (*n* = 264)	157 (75*–*378) (*n* = 258)	156 (82*–*461) (*n* = 276)
**Door to imaging, median minutes (IQR)**			
ESC	21 (13*–*25) (*n* = 30)	20 (15*–*29) (*n* = 43)	18 (14*–*27) (*n* = 36)
Transfers	22 (14*–*38) (*n* = 235)	23 (15*–*34) (*n* = 216)	25 (30*–*56) (*n* = 241)
Overall	22 (14*–*35) (*n* = 265)	22 (15*–*34) (*n* = 259)	24 (15*–*36) (*n* = 277)
**Door to thrombolysis, median minutes (IQR)**			
ESC	44 (23*–*57) (*n* = 17)	43 (29*–*60) (*n* = 25)	28 (24*–*37) (*n* = 16)
Transfers	54 (34*–*84) (*n* = 105)	50 (32*–*67) (*n* = 93)	49 (39*–*85) (*n* = 98)
Overall	50 (31*–*81) (*n* = 122)	48 (31*–*65) (*n* = 118)	42 (31*–*65) (*n* = 114)
**Door-to-groin puncture, median minutes (IQR)**			
ESC	64 (51*–*115) (*n* = 30)	65 (49*–*96) (*n* = 43)	51 (39*–*85) (*n* = 37)
Transfers	14 (9*–*28) (*n* = 265)	15 (9*–*26) (*n* = 250)	15 (16*–*38) (*n* = 282)
Overall	15 (9*–*32) (*n* = 295)	18 (10*–*38) (*n* = 293)	18 (10*–*34) (*n* = 319)
**Median symptom onset/last seen well to puncture, median minutes (IQR)**			
ESC	192 (119*–*497) (*n* = 40)	242 (125*–*698) (*n* = 50)	160 (102*–*555) (*n* = 44)
Transfers	335 (205*–*558) (*n* = 264)	376 (244*–*588) (*n* = 256)	355 (74*–*140) (*n* = 283)
Overall	322 (194*–*552) (*n* = 305)	349 (216*–*580) (*n* = 306)	347 (219*–*608) (*n* = 327)
**Puncture to recanalisation, median minutes (IQR)**			
ESC	26 (16*–*38) (*n* = 38)	22 (15*–*38) (*n* = 47)	22 (15*–*44) (*n* = 43)
Transfers	19 (13*–*28) (*n* = 250)	19 (12*–*28) (*n* = 239)	20 (16:49) (*n* = 275)
Overall	20 (13*–*29) (*n* = 288)	19 (13*–*29) (*n* = 286)	20 (13*–*33) (*n* = 318)
**Procedure duration, median minutes (IQR)**			
ESC	37 (25*–*61) (*n* = 40)	31 (24*–*52) (*n* = 50)	37 (18*–*57) (*n* = 43)
Transfers	31 (18*–*51) (*n* = 261)	26 (17*–*46) (*n* = 253)	27 (16*–*36) (*n* = 281)
Overall	32 (18*–*53) (*n* = 301)	27 (17*–*48) (*n* = 303)	28 (16*–*50) (*n* = 324)
**Symptom onset/last seen well to recanalisation, median minutes (IQR)**			
ESC	218 (141*–*500) (*n* = 38)	267 (145*–*464) (*n* = 47)	229 (113*–*603) (*n* = 43)
Transfers	371 (225*–*573) (*n* = 249)	409 (267*–*604) (*n* = 239)	380 (235*–*614) (*n* = 275)
Overall	348 (212*–*567) (*n* = 287)	375 (246*–*597) (*n* = 286)	375 (244*–*627) (*n* = 318)
**TICI 2b/2c/3 recanalisation**	91% (257/281)	94% (272/289)	96% (289/302)
**mRs 90 days post-thrombectomy**	≤ 2 = 45% (136/302)	≤ 2 = 45% (105/234)	≤ 2 = 48% (151/315)

## Discussion

The COVID-19 pandemic presented unprecedented challenges to modern healthcare systems. The time-sensitive nature of EVT for LVO stroke meant that these challenges were exacerbated with issues such as delays in patient presentation to hospital, staff, ambulance and bed shortages and new infection control protocols all having the potential to hinder service provision leading to delayed reperfusion and worse patient outcomes [23].

International studies from several countries including China, France, Germany and Spain have shown reductions in the number of EVT cases during the COVID-19 pandemic [23–26]. Reduced case numbers were experienced despite the known association between COVID-19 and ischaemic stroke with LVO, presumably related to delayed hospital presentations and delays in diagnostic and clinical assessment. For example, large multicentre studies performed by Zhoa et al. [23] in China and Kerleroux et al. [26] in France demonstrated 25.3% and 21% reductions in total thrombectomy cases performed during the COVID-19 pandemic, compared to equal time intervals before it.

The onset of the pandemic did not lead to a reduction in the number of EVTs performed at our institution with stasis in numbers between 2019 and 2020. In the years preceding the pandemic, there had however been a trend for year-on-year increases in EVTs, and the lack of such an increase in 2020 is therefore consistent with a “real world” reduction in numbers. This suggests that our initial hypothesis, that the COVID-19 pandemic would lead to a reduction in the number of EVTs performed, was correct. However, the relative maintenance of EVT numbers in the year after the pandemic onset, as well as the bounce in EVT numbers by 7% in the pandemic’s second year, is a testament to both the robustness of service provision at our ESC and at the referring hospitals. The increase in the number of EVTs performed in 2021 is all the more impressive given that it coincided with the commencement of a 24/7 thrombectomy service in Cork University Hospital, a factor that would have been expected to lead to reduced referrals to our service.

Contrary to our hypothesis that the pandemic would lead to delays in patient treatment, our findings demonstrated no major change to most intrahospital workflow time metrics pertaining to EVT. Median time from door to groin puncture, which constituted the primary outcome measure of this study, only slightly increased from 15 to 18 min between 2019 and 2020. Results are not dissimilar to previously published literature from other countries, including single- and multi-centre studies in Spain and Germany, which actually demonstrated small reductions in door-to-groin puncture times post-COVID-19 [24, 25]. In fact, for patients that presented directly to our ESC, this metric was observed to reduce by 14 min from 2020 to 2021 from 65 to 51 min. These findings most likely reflect increased efficiency associated with improving familiarisation with COVID-19 protocols.

With regards to the secondary outcome measures of this study, significant increases were observed in the median time from symptom onset/time last seen well to presentation and intervention. These delays in patient presentation are most likely explained by patient reluctance to attend hospital during the pandemic and are most pronounced in the group presenting directly to ESC, with median time to presentation from symptom onset increasing by over an hour between 2019 and 2020 (100 to 165 min). Interestingly, our experience of large delays between symptom onset in patients outside of hospital and presentation appears to be unmatched when compared to several European [26] studies. Rudilosso et al. [24] in Spain found ‘no difference in prehospital time metrics’ when comparing time from symptom onset to arrival at hospital in patients prior to and during the pandemic. Kerleroux et al. [26] in France reported similar findings, with no significant differences in time between symptom onset and imaging in patients with LVO between 2019 and 2020. Interestingly, significant time delays remain in 2021, despite continued relaxation of COVID-19 restrictions, with the median time from symptom onset to presentation being 57 min greater than in 2019 (157 min and 100 min).

The other time metrics which we examined in this study, such as median door-to-imaging time and median door-to-needle time were largely comparable across all study periods, with figures serving to emphasise the consistent and reliably prompt service provision in our ESC, despite the ongoing barriers against this from the COVID-19 pandemic.

Finally, this study also evaluated the impact of COVID-19 on procedural and patient functional outcomes following EVT. There was an excellent technical success rate of EVT across the study, with TICI2b-3 reperfusion rates in excess of 90% in all three time periods, improving from 91% in 2019 to 96% in 2021. Reassuringly, despite the marked delays in time from symptom onset to both presentation and institution of endovascular treatment from 2019 to 2020 and 2021, the proportion of treated patients who achieved a good functional outcome remained stable, with an mRS ≤ 2 seen in almost half of the patients managed with EVT in 2019, 2020 and 2021.

Although this is a retrospective study, all data was extracted from a prospectively maintained EVT database and the near complete data capture on almost 1000 patients is a huge strength. Data was only collected and assessed for patients referred to our service and therefore patients presenting to peripheral hospitals with strokes that were not referred to our service were not captured in this study. This means that the true impact of the pandemic on overall stroke numbers in Ireland has not been determined and further investigation is suggested.

## Conclusion

This study is the first paper evaluating the effect of the COVID-19 pandemic on EVT service provision in Ireland. Despite the myriad of challenges presented by the pandemic, service provision at the primary Irish ESC, and the referring hospitals, has proven to be robust. Procedural time metrics were maintained whilst the expected reduction in the number of EVTs performed did not materialise, there actually being a significant increase in the number of EVTs performed in the pandemic’s second year. Delays in patient presentation to hospital were identified; however, EVT success rates and patient outcomes did not appear significantly impacted.
